# Social Construction of the Value–Behavior Relation

**DOI:** 10.3389/fpsyg.2019.00934

**Published:** 2019-05-01

**Authors:** Vladimir Ponizovskiy, Lusine Grigoryan, Ulrich Kühnen, Klaus Boehnke

**Affiliations:** ^1^Bremen International Graduate School of Social Sciences, Jacobs University Bremen, Bremen, Germany; ^2^University of Bremen, Bremen, Germany; ^3^International Research and Teaching Laboratory of Sociocultural Research, National Research University Higher School of Economics, Moscow, Russia

**Keywords:** basic human values, behavior, attitudes, value instantiation, motivation, construal

## Abstract

Personal values are reliable cross-situational predictors of attitudes and behavior. Since the resurgence in research on values following the introduction of Schwartz’s theory of basic values, efforts were focused on identifying universal patterns in value–attitude relations. While some evidence for such universal patterns exists more recent studies point out, there is still considerable variation in value–attitude and value–behavior links across cultures and contexts. Extending the existing literature on potential moderators in this paper, we introduce the concept of value-instantiating beliefs. This study looks at subjective construal of the value relevance of specific behaviors as a proximal moderator of value–attitude and value–behavior relations. We argue that a belief that construes a behavior as a valid instantiation of a value is a prerequisite for the relationship between said value and the behavior. We also argue that such value-instantiating beliefs play a central role in determining the direction of the relationship. In a web-based survey experiment (*N* = 1724) consisting of three trials, we presented participants with vignettes describing behavioral choices. In order to manipulate the value-instantiating beliefs, the behaviors were described either neutrally, as reinforcing the value, or as inhibiting the value. We then measured the value-instantiating beliefs, the attitude toward the behavior, and the intention to perform it. Instantiating beliefs strongly moderated the relationship between the personal values and the dependent variables in all three trials. Moreover, the direction of the relationship was determined by the instantiating beliefs. The results emphasize the plasticity of the value–behavior relation and the role of social construction in directing the motivational power of values toward concrete instantiating behaviors.

## Introduction

Personal values are individual conceptions of the desirable that guide behavior (Schwartz, 1992) – in little things like donating to charity or spending time with the family and in life-defining decisions. In the abstract, people tend to agree on what is desirable, good and important in life: kindness, health, personal accomplishment, learning, fairness are all accepted as suitable end-goals without extensive deliberation. But when it comes to how abstract values translate into concrete behavior, historic and cultural context seems to play an important role.

Equality, for example, is a universal principle, appreciation of which we share with other, non-human primates: capuchin monkeys, famously, prefer to forgo food rather than accept unequal pay for their efforts (Brosnan and De Waal, 2003). While capuchins in the well-known experiment were able to observe each other perform the same task and receive different rewards, what constitutes equality in human societies is less obvious. For the larger part of human history, unequal participation of genders in economic and political life was seen as natural and justified (Engels, 2010). The view of traditional gender relations as violating the principle of equality has been brought about by scholarly and ethical arguments made in the 19th and 20th centuries (e.g., Beauvoir, 1972; Mill, 1997) Introduction of these arguments and subsequent public debate then formed new visions of equality that were understood and shared within communities, and this understanding of equality drove people’s sympathies and actions. Such differences in the ways values are understood and applied were extensively addressed within qualitative approaches to social science, for example, in the social representation theory (Moscovici, 1976), but fall outside the scope of contemporary values theory. The central starting point for the work that we present in this article is the notion that contemporary research on value–behavior relations largely ignores the role of social construction of specific attitudes. Therefore, we present a theoretical model that explicates the role of shared beliefs, specifically value-instantiating beliefs (VIBs) in the relationship between values and attitudes and present an experimental study that tests the core proposition of the model: that VIBs moderate the relationship between the value and the behavior.

### Theory of Basic Human Values

The concept of values has been used by academics at least since the early 20th century, and since then it was accompanied by definitional difficulties (Rohan, 2000). Values have been introduced to psychology and sociology as a concept rivaling social norms. Unlike social norms, values are not applied exclusively in specific situations, but are fundamental principles that are applicable to any situation or behavior, a more general principle that guides behavior. In most general sense, a value is something that is important to a person, a very abstract end state that he or she wants to achieve. The definition of the concept varies across authors, but it is generally agreed upon that values are (a) characteristics of individuals or groups, (b) conceptions of the desirable that (c) influence attitudes and behavior (Kluckhohn, 1951, p. 395).

**Figure 1:**
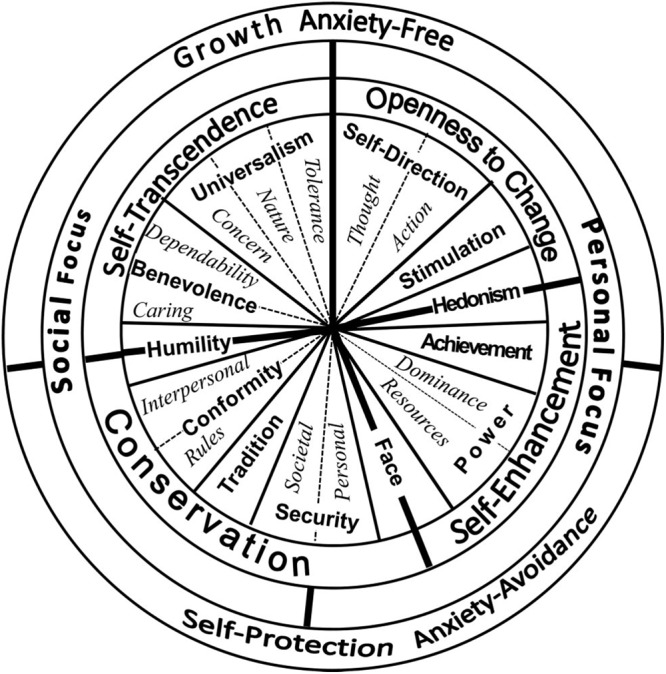
Motivational continuum of the basic human values. Adapted from Schwartz et al. (2012). Copyright 2012 by the American Psychological Association. Reproduced with permission.

Of course, each individual has a different set of preferred values, informed by the multitude of influences he or she has been exposed to during socialization (Schwartz and Bilsky, 1990). And yet, Schwartz and Bilsky (1990) developed a theory of how individual values are interrelated, which argues that there is a relatively simple, universal structure underlying individual value preferences. Based on earlier work Rokeach’s (1973), Schwartz (1992) focused on the motivational aspect of values, discerning two motivational dimensions along which the values are organized. The first dimension, labeled “openness to change versus conservation,” describes a conflict between openness toward change and new experiences on the one hand, and order, control, and restraint on the other. The second dimension, labeled “self-enhancement versus self-transcendence” relates to the conflict between the concern with the outcomes of one’s actions for the self versus the concern with these outcomes for the others. Within this motivational continuum, the original formulation placed ten, and the revised – 19 basic human values (Schwartz et al., 2012). According to Schwartz (1992), the boundaries between the basic values are arbitrary, and the value space can be partitioned in any suitable way. Values that are located close to each other share motivational goals, and are conceptually and functionally similar (Schwartz and Bilsky, 1990). The structure of relations among types of values is presented in Figure 1, and the conceptual definitions of the 19 basic human values are presented in Table 1.

**Table 1:** Basic human values and their conceptual definitions.

Value	Conceptual definitions in terms of motivational goals
Self-direction—thought	Freedom to cultivate one’s own ideas and abilities
Self-direction—action	Freedom to determine one’s own actions
Stimulation	Excitement, novelty, and change
Hedonism	Pleasure and sensuous gratification
Achievement	Success according to social standards
Power—dominance	Power through exercising control over people
Power—resources	Power through control of material and social resources
Face	Security and power through maintaining one’s public image and avoiding humiliation
Security—personal	Safety in one’s immediate environment
Security—societal	Safety and stability in the wider society
Tradition	Maintaining and preserving cultural, family, or religious traditions
Conformity—rules	Compliance with rules, laws, and formal obligations
Conformity—interpersonal	Avoidance of upsetting or harming other people
Humility	Recognizing one’s insignificance in the larger scheme of things
Benevolence—dependability	Being a reliable and trustworthy member of the group
Benevolence—caring	Devotion to the welfare of in-group members
Universalism—concern	Commitment to equality, justice, and protection for all people
Universalism—nature	Preservation of natural environment
Universalism—tolerance	Acceptance and understanding of those who are different from oneself

*Adapted with permission from Schwartz et al. (2012). Copyright 2012 by the American Psychological Association*.

An important implication of the organization of value types along the two motivational dimensions is the system of motivational conflicts among values. Behaviors that satisfy a motivational goal are likely to do so at the expense of the opposing value. When one engages in self-serving behavior, such as pursuit of wealth, it is likely to hinder the attainment of the pro-social motivational goal, and vice versa. This theorization has been tested in multiple countries (Schwartz, 1992, 1994), and the evidence is strongly in favor of near universal organization of values along the motivational dimensions.

The opposite ends of the two dimensions—the four motivational goals, designated by Schwartz et al. (2012) as higher-order value types, are important to human functioning and are valued by people universally. However, individuals differ in their value preferences: while most people would believe that helping close others is important and valued, helping others is often at odds with taking opportunities for oneself, and individuals vary in deciding what gets priority (Schwartz and Bardi, 2001).

Values, defined and measured as proposed by Schwartz, have been linked to various outcomes, including attitudes, beliefs, worries, personality traits, political preferences, consumer behavior, and other constructs (Grunert and Juhl, 1995; Thøgersen and Grunert-Beckmann, 1997; Boehnke et al., 1998; Roccas et al., 2002; Hitlin and Piliavin, 2004; Schwartz et al., 2010). Schwartz’s theory has been used widely in social psychological research and related fields, and the present study relies on Schwartz’s conceptual framework.

### From Values to Behavior

Values are typically seen as the organizing principles or determinants of attitudes, and behavior (Bem, 1970; Hitlin and Piliavin, 2004). Values are the source of motivation in the value–attitude–behavior model of value–motivated behavior (Feather, 1985; Homer and Kahle, 1988; Milfont et al., 2010). The relationship between values and behavior is mediated by value-relevant attitudes. For example, Homer and Kahle (1988) showed that values predicted attitudes toward natural foods, and these attitudes, in turn, affected shopping preferences. In another study (Feather, 1995), participants were presented with vignettes describing hypothetical situations with two alternative behavioral choices, where choices were expressive of different values. Values systematically related to the attractiveness of the choices, and to behavioral choices. However, values had no effect on behavioral choices when controlling for attractiveness.

Following the development of Schwartz’s value theory and its method of measuring personal values, correlational and causal dependency of attitudes on personal values has been demonstrated in studies of various attitudes, ranging from political attitudes to attitudes toward functional foods (see, for example, Maio, 2017 for a review).

An important aspect of the theorized hierarchical value–attitude relation is that attitudes are seen as expressions or subordinate consequences of values (Kristiansen and Zanna, 1991; Eagly and Chaiken, 1993). Attitudes can express values to a different degree (Maio and Olson, 2000), and while some attitudes are intuitively value-expressive – for example, attitude toward health insurance is likely to be related to the value of security – others can be less clearly linked to values. In the norm activation model, it was theorized that the effect of altruistic values on attitudes depends on the awareness of consequences of one’s actions for others (Schwartz, 1977); later studies supported this theorization (De Groot and Steg, 2009). In an experimental study, Maio and Olson (1995) demonstrated that “value-expressiveness” can be experimentally manipulated, affecting the strength of the value–attitude relation for self-transcendence as well as self-enhancement values.

Similarly to attitudes, behavior is systematically related to values. Individual differences in values have been shown to map to motivationally congruent behaviors across the value spectrum using both self-report and peer-reported measures of behavior (Bardi and Schwartz, 2003). In a meta-analysis of values and personality research, Fischer and Boer (2014) demonstrated a consistent relationship between values and behavioral dispositions in measures of personality. It is important to note that the effects of values on behavior are not strong, and there are many other, often more proximal, sources of variation in behavior (Maio et al., 2001).

Social psychologists previously argued for indirect pathways of the link between values and their enactment. Rokeach (1973) suggested that value-congruent behavior is motivated by need for consistency; it was also suggested that values might affect beliefs and personal norms, and, through them, behavior (Dietz et al., 2005). A recent review of neuroscientific literature on value–behavior relation, however, summarized evidence for a more direct link: Brosch and Sander (2013) suggested that individual value preferences may affect the worth that is given to different behavioral options in terms of perceived reward value.

### Moderators of the Effects of Values

Multiple studies emphasized the role of context in the relation between values and attitudes (e.g., Feather, 1985; Sagiv and Schwartz, 1995). While predicting readiness for outgroup contact from values, Sagiv and Schwartz (1995) derived and confirmed disparate hypotheses for the Jewish majority and Arab minority in Israel, arguing that the same object of attitude (outgroup contact) relates to different values in the studied contexts. In a different study, it was found that in countries where the relationship between the state and the church was amicable, expected positive relations between religiosity and values of conformity and tradition were present. However, in countries where church was in conflict with the state, religiosity correlated less strongly with conservation-type values, and more strongly with universalism (Roccas and Schwartz, 1997). More recently, it was found that the relationship between values and left–right political orientation reverses direction in post-communist countries as compared to countries with no history of communism. The authors argued that the differences in construal of the political spectrum in the studied countries could explain the findings (Barni et al., 2016).

A number of contextual moderators were proposed to explain differences in value–attitude links: salience of values could strengthen the value–attitude relation (Maio and Olson, 1995; Verplanken et al., 2008), salience of attitude could affect the strength of the relationship (Boer and Fischer, 2013), and the effects of the values on attitudes can be constrained in contexts with higher normative pressure on self-expression (Lönnqvist et al., 2006; Gelfand et al., 2011). These moderators, however, do not address the qualitative aspect of the value–behavior relation that we are interested in, i.e., do not relate to specific value–attitude and value–behavior pairings, focusing instead on general contextual conductivity for the effect of values.

The qualitative aspect of the relationship between abstract values and specific attitudes and behavior was elaborated in the work of Maio (2010). Maio argued that the representation of values varies in its abstractness, and that values are best understood as mental representations, or cognitive categories. Specific instances of these categories, then, are value-relevant behaviors and attitudes. Maio argued that, similarly to other subordinate elements of cognitive categories, value instantiations differ in typicality: Whereas ‘treating people equally regardless of their gender’ is a typical example of the value of equality, ‘treating people equally regardless of their right- or left-handedness’ is not. Experimental studies have shown that invoking typical as opposed to atypical instantiations of values affects behavior stronger (Maio et al., 2009).

Typicality of value instantiations was recently proposed as a moderator of value–behavior relations across cultural contexts. Hanel et al. (2017) reviewed evidence in support of the idea that certain behaviors can be typical representations of values in some countries, but not in others. For example, Hanel et al. (2017) found that while saving water was a typical instantiation of environmentalism in Brazil but not in United Kingdom, choosing environmentally conscious modes of transportation was a typical instantiation of the same value in United Kingdom, but not in Brazil. Hanel et al. (2017) hypothesized that values relate stronger to the typical compared to atypical instantiations of values.

Unlike other approaches, this conceptualization focuses on the properties of specific value–behavior relations. However, it inherits the essentialist understanding of the relationship between values and the behavior, where behavior represents a value due to the intrinsic qualities of the behavior. Typicality, then, reflects the degree to which a specific behavior corresponds to a central tendency for its superordinate cognitive category, the value: treating people unequally based on their race or height is equally unjust, yet discrimination by race is a more common example that possesses more archetypal features of injustice. Similarly to previously proposed moderators, the concept of typicality of instantiations allows us to explain the strength of the relationship between values and value-expressive attitudes and behavior: the relationship between values and their typical instantiations is hypothesized to be stronger relative to atypical instantiations.

### Value-Instantiating Beliefs as Potential Moderators of the Effects of Values: The Present Study

Several conceptualizations described above stressed the importance of context in the relationship between values and their outcomes. A few of these studies addressed contextual differences in meanings of attitudinal objects (e.g., Sagiv and Schwartz, 1995; Roccas and Schwartz, 1997; Barni et al., 2016). However, the implicit assumption that beliefs about attitudinal objects affect the relationship was left without elaboration. The particulars of construals were either hypothesized based on general knowledge (Sagiv and Schwartz, 1995; Roccas and Schwartz, 1997), or used *post hoc* to explain findings (Barni et al., 2016).

Additionally, previously proposed moderators explicitly addressed only differences in *strength* of the relationship between values and value-expressive attitudes and behavior. We, however, propose that there may be qualitatively different patterns of value–attitude and value–behavior relations depending on how the target objects of attitudes and behavior are construed. We postulate that the value–attitude relation becomes possible through individual beliefs that instantiate values. Our core proposition is as follows: a value can motivate an attitude toward a social object only if the individual believes that the attitude expresses the value. For example, for the value of universalism—nature to motivate preference for electric cars, one has to believe that electric cars are more environment-friendly than the alternatives. If a person believed that choosing an electric car would harm the environment, their value of universalism—nature would motivate dislike toward electric cars.

On the group level, if a belief is shared among the majority of individuals, samples drawn from the population will show a statistical relationship between the value and the attitude. If the majority believes that electric cars are environment-friendly, those for whom protecting the environment is an important life priority will have more positive attitudes toward the electric cars than those for whom it is less important. If, however, the belief is not shared to a certain degree, such statistical relationship would become impossible.

This property, the ability to moderate value–attitude relations, is what sets beliefs that link social objects to values apart from other beliefs. We call such beliefs VIBs. Examples of VIBs are “having a pet is responsible,” “this car is safe” or “eating meat is murder.” VIBs acquire motivational properties from values. Most beliefs on their own do not compel us to act, but VIBs do: if we value safety, we avoid things that we believe are unsafe and pursue those that we believe to be safe; if we value power, we pursue things that we believe can make us rich. Without VIBs, the relationship between values and attitudes is impossible: if we believe that nothing is safe or unsafe, the value of security loses its motivational power.

Value-instantiating beliefs can differ in strength (one can believe that a car is relatively safe or extremely safe) and in direction (a person can believe that a car is rather safe or rather unsafe). These properties of VIBs would determine the strength and the direction of the value–attitude relation. VIBs refer to a specific object and to a specific value.

Value-instantiating beliefs are not equally distributed among individuals and among groups. Some people believe that support for immigration is dangerous for themselves or the society, and are motivated to oppose immigration by their value of security. Others, who do not see immigration as threatening, might support it even if security is very important for them. Likewise, it is not difficult to imagine differences in VIBs across communities: in the mainstream society, vaccination against polio may be considered an effective way to protect personal health, but members of the anti-vaccination movement believe that vaccination is dangerous. Such differences in VIBs would bring about differences in co-occurrence of values and attitudes within those groups. In the general population, the value of security—personal can be positively related to the attitudes toward vaccination, but among the vaccination skeptics, those who hold personal security more important might have stronger negative attitudes toward vaccination.

At this point, there is a need to situate the VIB vis-à-vis a related construct, the social representation. Social representations are similar to VIBs in that they are used by individuals to evaluate and act upon objects in their social surroundings (Moscovici, 1973). Similarly to VIBs, social representations are specific to particular contexts (Moscovici, 1973), and are often contested and negotiated (Howarth, 2006). Compared to social representations, VIBs are narrow in scope: social representations encompass all values, ideas, and practices related to the target, while VIBs are singular beliefs that relate the target to a specific value. Unlike social representations, VIBs are individually held beliefs that may be shared to a different degree. Interpersonal variation in VIBs may be useful for explaining within-group variability in motivation toward specific behaviors, while intergroup differences might explain differences in coordinated behavior. Unlike social representations, VIBs are summary judgments. The same individual can hold multiple or even competing social representations on the same subject, but a person can hold only one corresponding VIB. For instance, a person may be aware of both traditional and feminist discourses on gender relations, but can only have one summary belief about the fairness of traditional gender roles. We argue that on individual level, VIBs can be strongly informed by social representation of the corresponding social object. On the group level, shared VIBs approximate the value aspect of the corresponding social representation, with aforementioned caveats.

This study aims to provide initial evidence for the utility of VIB as a construct and for its moderating role in value–behavior relations. The scope of the study is limited to establishing the theoretical mechanism that links construal of social objects to value-based motivations for engaging with them. The questions of VIB acquisition and change on a broader societal level remain outside the scope of the study and open to future investigation.

In an experimental study, we attempted to manipulate the VIBs by varying the quantity of information about consequences of a behavior for values. We presented participants with value-relevant behavioral choices. To minimize interference from existing construals, we developed novel, hypothetical stimuli. Across experimental conditions, we described the target behavior as either reinforcing the value, as thwarting the value, or neutrally. Based on the above theorization, we developed the following hypotheses, ranging from weak to strong.

H1: VIBs will moderate the relationship between the value and the attitude toward the behavior, and between the value and the behavioral intention.

H2: Positive VIB manipulation will produce a more positive relationship between the value and the dependent variables compared to control and to the negative manipulation conditions, and the negative manipulation will produce a more negative relationship than the other two conditions.

H3: Positive VIB manipulation will result in a positive relationship between the value and the dependent variables, and the negative manipulation will result in a negative relationship.

The research question, hypotheses, design, sample size, and exclusion criteria were preregistered via an AsPredicted form: https://aspredicted.org/x7h46.pdf.

## Materials and Methods

### Participants

Sample size was determined based on a power analysis performed with GPower software (Faul et al., 2007) with power (1 – β) set at 0.95 and α at 0.05. Since we did not predict a specific effect size, we based our power calculation on *f*^2^ = 0.009, an average effect size of an interaction in psychological literature (Aguinis et al., 2005). The analysis indicated *N* = 1614 needed to detect an interaction effect.

We included the following exclusion criteria in the preregistration: participants were to be removed from the study if they failed two out of two attention check questions, or if they provided the same answer for 50 or more out of the 57-item measure of values.

A total of 1,867 participants were recruited via Amazon Mechanical Turk. One hundred and fourteen participants dropped out of the study before completing the experimental part, and 29 were excluded from the analyses in agreement with the exclusion criteria, resulting in an effective sample size of 1,724 participants. Among the participants, 48.4% identified their gender as male, and 51.4% as female. Mean age was 39.5 years (*SD* = 12.7), and 63.4% held Bachelor’s degree or higher. 82.2% of participants indicated the United States as their country of residence, 14.3% resided in India, and 3.5% listed a different country as a country of residence.

### Design and Procedure

The study was performed as a web-based survey experiment. Before data collection, informed consent was obtained from all participants. In the first part of the survey, we obtained sociodemographic variables and assessed individual value preferences. Sociodemographic assessment included measures of age, gender, level of education, country of residence, and level of religiosity. These variables were shown to relate to values and to value-relevant attitudes in prior research (Schwartz and Rubel, 2005; Schwartz, 2006, 2012b; Davidov et al., 2008). The experimental part of the study consisted of three trials. Each of the trials examined the relationship between a specific value from Schwartz’s refined theory of basic human values and a behavior under three conditions: a positive VIB condition, a negative VIB condition, and control. The first trial examined the value of universalism—concern, the second – security—personal, and the third – conformity—rules. These values were selected because they have been shown to relate to a range of socially relevant outcomes, such as attitudes to minorities, voting behavior, consumer choices, and others (see Schwartz, 2006, for a review), and because they represent both motivational dimensions of the theory of basic human values.

In every trial, the participants were asked to read a vignette describing a hypothetical situation involving a behavioral choice. Within each trial, participants were randomly assigned to one of the three conditions, each receiving a slightly modified version of the vignette: in the positive VIB condition, the vignette described the behavior as promoting the value, in the negative VIB condition the same behavior was described as inhibiting the value, and in the control condition no value-related statements were explicitly made. Information in the descriptions was not mutually exclusive across conditions.

In the first trial (universalism), the vignette described a male applicant for an IT position who is an immigrant. The control condition included information only about the applicant’s good fit for the job and his immigration background. In the positive VIB condition, the applicant’s experiences with discrimination on the labor market were also described. We reasoned that hiring a member of a discriminated minority is compatible with the motivational goal of universalism—concern (protection of the weak members of society, equality, and justice). In the negative VIB condition, the description of labor market discrimination was replaced with a description of sexual harassment accusations from the applicant’s former employee. Participants’ attitude toward hiring the person and intention to hire the person were measured.

In the second trial (security), participants were presented with a scenario where they had to arrange a ride with a stranger through a ride sharing website. The control condition simply stated that the driver under consideration has mostly positive, but also several negative reviews on the website. In the experimental conditions the content of the reviews was manipulated. In the positive VIB condition, the driver was described as “extremely careful” and “confident.” In the negative VIB condition, it was mentioned that the driver was young and made a lot of stops to have some energy drinks, as “it seemed like he partied before the trip.” The attitude and behavioral intention to share the ride with this driver were measured.

Finally, the third trial (conformity) described a professional development course and a hypothetical colleague’s descriptions of the course were manipulated. In the positive VIB condition, this colleague mentioned that the course instructor requires complete trust in her and her method and expects students to memorize and reproduce the material. In the negative VIB condition, the instructor was asking challenging questions and did not shy away from controversial topics. In the control condition, no value-specific information was provided. The attitude and behavioral intention to enroll in the class were measured.

The full texts of the vignettes can be found in Appendix A. After each trial, the participants answered a manipulation check question, followed by measures of attitude and behavioral intention. On average, it took the participants 10 min and 41 s to complete the study. Following the three trials, participants were debriefed about the purpose of the study and provided with contact details of the principal investigator.

### Measures

Individual value preferences were 1assessed using the Portrait Values Questionnaire—Revised (PVQ-RR, Schwartz, 2017). PVQ-RR assesses 19 values from the revised formulation of the theory of basic human values using 57 items, three for each value. Items describe value preferences of fictitious persons, and participants are asked to indicate how similar they are to the described person (from 1—not like me at all to 6—very much like me). Scores for individual values were computed by averaging the responses for the items assessing each value. These scores were then centered on the mean of each respondent to compensate for differences in the use of the scale (Schwartz, 2003). Although mean-centering is conventional in value research, some recent studies criticize this approach (He and Van De Vijver, 2015; Borg and Bardi, 2016). To make sure that the findings are robust, we also run the tests with raw, uncentered value scores. The results of these tests are presented in Appendix B.

The measures of attitudes and behavioral intentions were developed in accordance to the guidelines by Armitage and Conner (2001). Attitude toward the behavior was assessed using a 7-point semantic differential with anchors “harmful” — “beneficial,” “good” — “bad,” “pleasant—unpleasant,” and “worthless” — “useful.” The reverse-scored items were recoded so that higher scores indicated more positive attitude toward the behavior. Reliability of the measure was assessed separately for each condition within each trial, resulting in nine scores for Cronbach’s α that ranged from 0.78 to 0.90 (*M* = 0.84).

Three items were used to measure behavioral intentions: “I would expect to [perform the behavior],” “I would want to [perform the behavior],” and “I would intend to [perform the behavior].” The scores for these measurements ranged from 1 to 7 (1—strongly disagree, 7—strongly agree). Cronbach’s α for the behavioral intention measure ranged from 0.92 to 0.96 (*M* = 0.95) across trials and conditions. Pearson correlations between measures of attitude and behavioral intention ranged from 0.65 to 0.81 (*M* = 0.73).

Obtained sociodemographic characteristics included age, gender, country of residence, education level, and degree of religiosity. Level of education was assessed using an ordinal scale with responses indicating the highest completed level of education (0—no high school, 1—high school diploma, 2—some college, no degree, 3—Associate degree, 4—Bachelor’s degree, 5—Master’s degree, 6—Professional degree, and 7—doctorate). Degree of religiosity was measured with a question “Regardless of whether or not you belong to a particular religion, how religious would you say you are.” The scores ranged from 1 to 10 (1—not at all religious, 10—very religious).

The effect of manipulation was assessed using a single item. Participants were asked “how just,” “how safe,” and “how disciplined and organized” would it be to perform the behavior described in the vignette. The responses were recorded on a 7-point Likert-type scale.

## Results

First, we compared manipulation checks to assess whether manipulations worked as intended. In the first trial describing an immigrant applicant, participants assessed hiring this person as less just in the negative VIB condition (*M* = 2.74) compared to the control condition (*M* = 5.87), *p* < 0.001. Positive VIB condition (*M* = 5.74) did not differ from the control, *p* = 0.221. In the second trial describing a driver for ride sharing, participants assessed sharing the ride as less safe in the negative VIB condition (*M* = 3.80) compared to the control condition (*M* = 4.18), *p* < 0.001, and as more safe in the positive VIB condition (*M* = 5.84), *p* < 0.001. In the third trial describing a professional development course, the course was assessed as less disciplined and organized in the negative VIB condition (*M* = 5.16) compared to the control (*M* = 5.73), and in the positive VIB condition – more disciplined and organized (*M* = 6.18) compared to the control, both tests significant at *p* < 0.001. Overall, the manipulation checks performed as predicted in all trials with the exception of Trial 1, where the difference between the positive VIB and the control conditions was not significant. The effects sizes for manipulation checks were large in Trial 1 (η^2^ = 0.53) and Trial 2 (η^2^ = 0.32) and medium in Trial 3 (η^2^ = 0.13).

Further, we compared the groups in each of the trials on the socio-demographic variables: Age, gender, education, religiosity, and country of residence. Only one difference was significant: in the first trial, participants in Condition 1 (positive VIB) scored significantly lower on religiosity (*M* = 4.63, *SD* = 3.29) than participants in Condition 2 (negative VIB) (*M* = 5.33, *SD* = 4.96).

**Figure 2:**
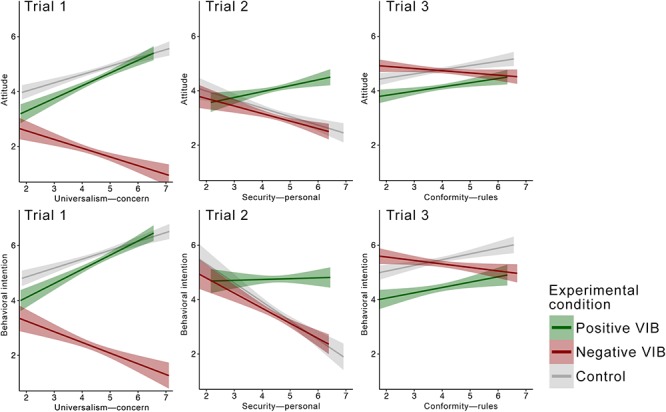
Effects of value on attitude and behavioral intention in the three trials. OLS regression lines were calculated separately for each condition. Shaded bands represent 95% CI.

To test our hypotheses, we performed a series of analyses of the relations between values and the dependent variables across conditions in the three trials. The patterns of relations are presented in Figure 2.

To assess Hypothesis 1, we conducted analyses of covariance for each of the three trials. For the purpose of the analysis, attitude and behavioral intention were dependent variables, and condition, value, and the interaction between value and condition were predictors. Since participants in the first Trial differed in religiosity across conditions, we included religiosity as a control variable in the model for that trial.

The results are summarized in Table 2. All six interaction terms had significant effects on the DV’s, providing support for Hypothesis 1. Following the rules of thumb given by Miles and Shevlin (2001), we can say that in Trials 1 and 2 the effects on both the attitude and the behavioral intention were large, and in Trial 3—medium.

**Table 2:** Summary of analyses of covariance.

	Attitude			Behavioral intention		
	*F*	*p*	$\eta_{p}^{2}$	*F*	*p*	$\eta_{p}^{2}$
**Trial 1**						
Intercept	280.1	< 0.001	0.153	324.04	< 0.001	0.173
Religiosity	2.10	0.148	0.001	11.6	0.001	0.007
Value (UNc)	20.9	< 0.001	0.013	20.0	< 0.001	0.013
Condition	4.41	0.012	0.006	4.23	0.015	0.005
Value^∗^condition	44.7	< 0.001	0.054	48.2	< 0.001	0.058
**Trial 2**						
Intercept	427.1	< 0.001	0.202	518.6	< 0.001	0.235
Value (SEp)	8.66	0.003	0.005	54.2	< 0.001	0.031
Condition	5.66	0.004	0.007	5.83	0.003	0.007
Value^∗^condition	16.5	< 0.001	0.019	16.3	< 0.001	0.019
**Trial 3**						
Intercept	1336.2	< 0.001	0.438	971.4	< 0.001	0.361
Value (COr)	7.54	0.006	0.004	6.27	0.012	0.004
Condition	15.7	< 0.001	0.018	18.1	< 0.001	0.021
Value^∗^condition	8.44	< 0.001	0.010	9.64	< 0.001	0.011

*UNc, universalism—concern; SEp, security—personal; COr, conformity—rules*.

In testing Hypothesis 2, we looked at the interactions between the value and each condition in predicting attitude and behavioral intention using control condition as a reference group.

In the vignette describing the immigrant IT applicant, as we expected, the effect of universalism—concern on attitude toward the behavior in the positive condition was significantly more positive (*b* = 0.161, *t* = 2.03, *p* = 0.043) and the slope in the negative condition significantly more negative (*b* = -0.626, *t* = -7.48, *p* < 0.001) than in the control condition. Similarly, the effect of universalism—concern on the intention to hire was significantly more positive (*b* = 0.193, *t* = 2.17, *p* = 0.030) in the positive condition, and significantly more negative (*b* = -0.727, *t* = -7.73, *p* < 0.001) in the negative condition.

In the vignette describing the ride-share driver, the effect of security—personal on the attitude was significantly more positive in the positive condition (*b* = 0.529, *t* = 5.09, *p* < 0.001), but the effect of the value on the attitude did not differ between the negative and the control conditions (*b* = 0.035, *t* = 0.34, *p* = 0.734). Likewise, the effect of security—personal on the intention to take the ride was significantly more positive in positive condition (*b* = 0.726, *t* = 5.35, *p* < 0.001), but slopes in negative and control conditions did not differ (*b* = 0.140,*t* = 1.03, *p* = 0.302).

In the vignette describing the professional development course, the effect of conformity—rules on the attitude was not different between the positive and the control conditions (*b* = -0.002, *t* = 0.72, *p* = 0.974), but the effect was significantly more negative in the negative compared to the control condition (*b* = -0.242, *t* = -3.50, *p* < 0.001). The effect of conformity—rules on intention to take the course did not differ in the positive compared to the control condition, but in the negative condition the slope was significantly more negative compared to control (*b* = -0.342, *t* = -3.80, *p* < 0.001). Eight out of 12 hypothesized relations turned out as predicted. We interpret these findings as mixed support for Hypothesis 2.

To test Hypothesis 3, we performed simple slope analyses as summarized in Table 3. The direction of the relationships was as predicted for all 12 slopes. Eleven out of 12 relations were significant^1^.

**Table 3:** Simple slope analyses for the effects of value on attitude and behavioral intention in each experimental condition.

	Attitude		Behavioral intention	
	*b*	*SE*	*b*	*SE*
**Trial 1**				
Positive VIB	0.46***	0.06	0.52***	0.07
Negative VIB	-0.32***	0.07	-0.39***	0.09
Control	0.30***	0.05	0.32***	0.05
**Trial 2**				
Positive VIB	0.22**	0.07	0.031	0.09
Negative VIB	-0.28***	0.08	-0.56***	0.10
Control	-0.31***	0.07	-0.70***	0.10
**Trial 3**				
Positive VIB	0.16**	0.05	0.20**	0.08
Negative VIB	-0.08*	0.05	-0.13*	0.06
Control	0.16***	0.05	0.21***	0.06

*Statistical significance levels: ^∗^*p* < 0.05, ^∗∗^*p* < 0.01, ^∗∗∗^*p* < 0.001*.

## Discussion

We set out to assess the effect of behavior construal on the value–attitude and value–behavior relationship. Taken together, our findings support that proposition that value–behavior relations are malleable, and that VIBs play an important role in the translation of values into specific attitudes and behavior. We found support for the strongest version of our hypotheses (H3): manipulation of behavior construal not only affected the strength of the relationship between values and behavior, but determined the direction.

Contrary to our hypotheses, values were systematically related to attitudes and behavior even when no value-expressive arguments for behavior were introduced. Furthermore, the effects in control conditions were of similar magnitude compared to the experimental conditions. We interpret this as indication of transitive property of VIBs. While we attempted to provide participants with novel, unfamiliar behavioral options to avoid carry-over effects from previously held VIBs, apparently, participants still interpreted situations according to the types of situations that they represented. For example, sharing a ride with a stranger was construed as unsafe even though no information about (un-)safety of that particular action was deliberately provided. Arguably, participants relied on their beliefs about trusting strangers in different situations.

### Implications for the Theory of Basic Human Values

The starting proposition of Schwartz’s theory of basic human values is that values are cognitive representations of universal evolutionary-based motives: needs of individuals as biological organisms, the need for coordinated social interaction, and the need for survival and well-being of social groups. Yet, in specific circumstances, the same evolutionary needs might be served by different actions: in many parts of the world, personal health is still best protected by procuring sufficient food, while in the economically developed countries overeating is now a more salient health risk. Social standards according to which we judge personal success vary across contexts. Things that were considered socially just and fair in the past are not anymore. Our findings explicate and formalize a previously unaddressed assumption that context-specific beliefs about social objects shape value-based motivation toward these objects in specific historical and cultural circumstances.

Our findings are also consistent with the differentiation of the levels of abstraction at which values operate as proposed by Maio (2010): while values can be viewed as a reflection of the universal motivational continuum at the most abstract level, they can also be seen as relatively independent cognitive categories that are meaningfully linked to other mental representations. This also addresses an early criticism levied against the cross-cultural research on values: that the meaning or content of values may vary across cultural contexts (Peng et al., 1997). While values show remarkable consistency on the most abstract level of representation (Cieciuch et al., 2017), their particular instantiations can be significantly affected by context-specific construals.

### Implications for the Research on Value–Attitude and Value–Behavior Relations

We have formalized and assessed the assumption that the effect of values on attitudes and behavior is contingent on beliefs about the target behavior or attitude. The theorization presented in this article advances previous work on consequences of values in the following ways.

First, we introduced a strong moderator of the link between values and their correlates, the VIB. It is a proximal moderator that may underlie some of the previously studied moderating effects on the value–attitude relations, such as salience of value, salience of attitude, and specific contextual moderators. It was argued that values are likely to affect attitudes and behavior stronger if the value or the attitude is salient in context (Maio and Olson, 1995; Verplanken et al., 2008). From our perspective, on the individual level, activation of a value is likely to increase the accessibility of VIBs pertaining to that particular value. On the group level, a value that is contextually salient, such as security at time of war, is likely to occupy a central place in the discourse. Public statements and private conversations will prominently feature statements linking various behaviors to the salient value, forming or increasing the salience of existing VIBs. Similarly, attitudes that are salient in a particular context are likely to be discussed in relations to values, strengthening the relation between values and attitudes through relevant VIBs. The VIBs are easier to assess than salience of values or attitudes in a social context, are applicable across situations, and may aid in hypothesis development for studies of consequences of values. It is worth noting that the VIB is distinct from a related moderator of value–behavior relations, the typicality of value instantiations (Maio et al., 2009; Hanel et al., 2017). In the experiments of Maio et al. (2009), presenting participants with a typical instantiation of a value (discrimination based on gender) produced a stronger effect on subsequent discriminatory behavior compared to an atypical instantiation (discrimination based on left-handedness). Yet, there was no disagreement between participants on whether the two types of discrimination instantiated the value of equality, i.e., whether they were unfair. Furthermore, both behaviors were perceived as equally unfair, suggesting similar VIBs pertaining to both behaviors. We could speculate that the cognitive property of typicality of value instantiations can be related, on the social level, to the “thickness” of discourse surrounding the VIB, or to cognitive support for it. Gender equality remains a prominently featured topic in contemporary discourse on social justice, while bias against left-handed people is much less discussed. The proportion of value-related discourse that is occupied by a specific instantiation may make it more likely that this instantiation is processed as typical. This speculation, however, was not tested in the present study.

Second, the proposed conceptualization provides a theoretical framework for incorporating questions of social construal of behavior into quantitative research on value–behavior relations. The questions of meaning-making and negotiation traditionally fall in the milieu of constructivist approaches to social sciences, such as the social representation theory, and were often addressed with qualitative methods. At the same time, these questions are particularly relevant for research on values that is firmly rooted in the mainstream, post-positivist approach to social psychology. The construct of VIBs can help bridge this gap by bringing social construction within reach of survey and experimental methodology.

Third, in certain cases, VIBs may be useful for addressing construct bias in cross-cultural research. It is often assumed that a certain behavior serves different functions across cultures. For example, obligations toward elder relatives are thought to be different in Western and non-Western contexts, making assessment of constructs such as familial piety problematic (Van de Vijver and Tanzer, 2004). However, the specific functions are rarely assessed. The VIB may be useful in measuring the equivalence of value-expressive functions of behavior, e.g., the degree to which specific familial obligations are construed in terms of the values of tradition or benevolence.

### Limitations and Future Directions

One may wonder to what extent can we consider the behavioral scenarios as being equivalent, and, consequently, to what extent are the measures of our dependent variables comparable across conditions. After all, sharing a ride with a person that we see as reliable is not the same as entrusting oneself to a dangerous driver.

The point we are trying to argue is that the same behavior and the same object of attitude can be, and is, seen in different light by different people. People are exposed, and expose themselves to different sources of information, forming their realities through the specific construals they acquire. During the recent “migrant crisis” in Europe, for example, consumers of the left-wing media have likely exposed themselves to representations of migrants as victims of discrimination and unfair treatment, while populist movements in Europe and abroad painted the migrants as recipients of unearned privileges and a security threat. Even if both representations relied solely on accurate factual information for support, the value-related arguments and beliefs that are formed through them are dramatically different. Yet, attitudes toward migrants are commonly assessed by social psychologists without accounting for these crucial differences in construal – a situation that our conceptualization aims to aid.

More formally, we can argue the same point using Searle’s (1995) theory of social construction. Searle differentiated between brute facts that exist independently from observers (such as that Everest is 8,848 m high), and institutional facts, that exist only insofar as people agree about them. Institutional facts, in Searle’s formulation, take the form of “object X stands for function Y in context C.” For example, “bills issued by the Bureau of Engraving and Printing (X) count as money (Y) in the United States (C)” (Searle, 1995, p. 28). X can be a brute fact or a function, allowing institutional facts to build upon each other, for example “money counts as a taxable asset if it is received as a salary.” Expanding this conceptualization, the function of social objects can be not only utilitarian, but also value-expressive, for example “money is the root of all evil in the United States.” Alternative value-expressive function that could be assigned to the same object could be “money is the sign of God’s grace in the United States.” Here, disagreement over what is the meaning of money in a particular context does not imply a disagreement over what is money: these institutional facts exist on the different levels of the hierarchy of social construction. Similarly, in our experiment we manipulated the value-expressive function of behaviors, but did not change the constitutive part of the behavior. We also took precautions to avoid mutually exclusive factual statements in different versions of the vignettes.

The use of online self-reported measures of the attitude and the behavioral intention has certain limitations. While prior studies showed that the values affect self-reported and peer-reported behavior in similar ways (Bardi and Schwartz, 2003), attitudes and intentions can be problematic predictors of actual behavior (see Fischer, 2017 for a review). Intentions for action in imaginary scenarios are also less amenable to many situational factors that affect behavior, such as social norms, perceived behavioral control, and others. However, the VIBs most likely moderate the value–attitude link of the value–attitude–behavior hierarchy. The findings pertaining to the attitudes and to the behavioral intentions differed minimally, supporting this reasoning. Nevertheless, a conceptual replication of our findings that would use measures of actual behavior could help gauge the relevance of differences in construal for enacted behavior.

In this study, VIBs were manipulated across conditions. A questionnaire measure of VIBs that would yield comparable scores across contexts could help extend our conceptualization to studies with correlational designs for use in contexts where experimental manipulations are not feasible. While measuring beliefs is relatively straightforward, there are several difficulties to resolve. First, VIBs pertain to specific values-as-categories, and not to the motivational continuum as described by Schwartz. Thus, using value definitions from the theory of basic human values might be insufficient. While the value of creativity is motivationally close to the value of Self-Direction—Thought (Schwartz, 2012a), and we expect people to believe that writing poetry is an instantiation of creativity, asking people if writing poetry is a way to “figure things out themselves” or to “form their views independently” is likely to result in confusion. Second, measures of specific values demonstrate much lower cross-context comparability compared to the underlying motivational dimensions (Davidov, 2010). Finally, ideally, the measure should incorporate an assessment of typicality of and cognitive support for the VIB, which are conceptually harder to assess.

Should these matters be resolved, we expect measurement of VIBs to be especially potent in explaining differences in value-relevant behavior across societies that are exposed to different discourses on the same issue (for example, on immigration, religion or political attitudes). It can also be relevant to the study of controversial behaviors and attitudes that divide societies, and to modeling cultural change. However, it is important to note that lack of differences in construal does not imply the lack of construal: even in contexts where people agree upon what value-expressive meaning is assigned to behaviors, such assignments still underlie the motivational effect of values on attitudes and behavior. Our conceptualization provides opportunities for experimental and observational cross-cultural research. What proportion of differences in value-motivated behavior can be explained by differences in values compared to differences in VIBs? What is the role that value-related discourses play in cultural change? Do changes in VIBs follow the changes in economic reality of societies?

Another intriguing avenue is the application of value instantiation research to acculturation. How does the acquirement of new VIBs happen following immigration? To give a couple of concrete examples, when does a person arriving in the United States begin to view jury duty as a representation of civic duty? How are more progressive VIBs regarding homosexuality acquired? How do individuals co-manage VIBs formed in interaction with home country and receiving country discourses?

## Conclusion

Personal values are universal principles that guide behavior. Yet, specific ways in which values are enacted differ across cultural and historic contexts. Building on existing literature on value instantiation, we present a novel framework that accounts for the social construction of the value-expressive function of behavior. In three experimental trials, we demonstrate the plasticity of the value–behavior relation under the effect of construal of specific behaviors. The new conceptualization provides opportunities for research on the social underpinnings of the value–behavior effects and the role of public discourse in directing value-expressive behavior.

## Ethics Statement

All subjects gave written informed consent before participating in the study in accordance with the Declaration of Helsinki. The data was collected in accordance with the German Research Foundation’s Guidelines for good scientific practice. The study did not require approval by the ethical review board as per national regulations and Jacobs University Bremen guidelines.

## Author Contributions

VP conceived of the original idea. VP and LG developed it into the presented theorization. VP, UK, and KB developed the method and the analytic strategy. VP managed the data collection. UK and KB supervised the project. All authors have participated in the preparation of the final manuscript, read and approved the final version.

## Conflict of Interest Statement

The authors declare that the research was conducted in the absence of any commercial or financial relationships that could be construed as a potential conflict of interest. The reviewer GM and handling Editor declared their shared affiliation and their involvement as co-editors in the Research Topic, and confirm the absence of any other collaboration.

## Footnotes

1 The results when using the raw scores for values were similar to those presented here. See Appendix B for more details.
