# Supplementary material for: Social Construction of the Value–Behavior Relation
Source: Front Psychol. 2019 May 1;10:934. doi: 10.3389/fpsyg.2019.00934 (PMC6504687; doi:10.3389/fpsyg.2019.00934)
Supplement: Supplementary file 1 [file Data_Sheet_1.docx]

Appendix A

**Social Construction of the Value-Behavior Relation**

Vladimir Ponizovskiy*, Lusine Grigoryan, Ulrich Kühnen, Klaus Boehnke

*** Correspondence:** Vladimir Ponizovskiy: ponizovskiy@bigsss.uni-bremen.de

Vignettes for the experimental conditions

**Trial 1, control condition**

Please read the following excerpt carefully:

Imagine that you are in charge of the hiring process at a company. You have interviewed a person who fits the requirements for the position of a senior IT specialist. The applicant’s name is Ege Yalcin. He has substantial experience working in a position of a supervisor of an IT department in a foreign country. During the interview, the applicant demonstrated good understanding of the tasks he will be charged with, and his language skills were sufficient for the job.

You have no applicants that would be equally fit for the job at this point, but you have two more weeks to fill the position and may keep looking.

**Trial 1, Positive VIB condition**

Please read the following text carefully:

Imagine that you are in charge of the hiring process at a company. You have interviewed a person who fits the requirements for the position of a senior IT specialist. The applicant’s name is Ege Yalcin. He has substantial experience working in a position of a supervisor of an IT department in a foreign country. During the interview, the applicant demonstrated good understanding of the tasks he will be charged with, and his language skills were sufficient for the job.

During the interview, the applicant told you that it was difficult to find a job after he immigrated. While he got numerous invitations for interviews, many of those interviews were cut short after he disclosed that he was an immigrant. One human resources officer told him that, in her opinion, hiring a foreign national would be too complicated from the legal standpoint – you know that in reality that is not the case. He was hired once by a small IT firm, but was later fired over a disagreement with a coworker.

You have no applicants that would be equally fit for the job at this point, but you have two more weeks to fill the position and may keep looking.

**Trial 1, Negative VIB condition**

Please read the following text carefully:

Imagine that you are in charge of the hiring process at a company. You have interviewed a person who fits the requirements for the position of a senior IT specialist. The applicant’s name is Ege Yalcin. He has substantial experience working in a position of a supervisor of an IT department in a foreign country. During the interview, the applicant demonstrated good understanding of the tasks he will be charged with, and his language skills were sufficient for the job.

Before the interview, you contacted the applicant’s last employer. They told you that they had to fire the person because of blatant workplace sexism. They told you that the applicant made sexual jokes about female coworkers and had to be moved to a division run by a male after failing to take direction from a female superior. The applicant was fired after an incident that could be described as physical harassment. Your IT department has a relatively even gender balance.

You have no applicants that would be equally fit for the job at this point, but you have two more weeks to fill the position and may keep looking.

**Trial 2, control condition**

Please read the following text carefully:

You have to travel long distance next weekend, and the only option to get to your destination is by arranging for a ride with a stranger going the same direction over the Internet.

You create an account on a ride sharing website and find that there is a person going in the desired direction at an acceptable time. The driver’s name is Ian, and he is driving a sedan car. His account has mostly positive reviews, but also several negative ones.

You do not have other options at this time, but several new rides appear on the website each day, and you have several days to keep looking.

**Trial 2, positive VIB condition**

Please read the following text carefully:

You have to travel long distance next weekend, and the only option to get to your destination is by car sharing – arranging a ride with a stranger going the same direction over the Internet. You register for an account on a ride sharing website and find that there is a person going in the desired direction at an acceptable time.

The driver’s name is Ian, he drives a Volvo sedan. He has mostly positive reviews on his account, for example:

“Ian is an extremely careful, confident driver, and he keeps his car in excellent condition. Drive with Ian!"

But also some negative reviews, such as:

“Ian drove well below the speed limit and took breaks to rest. Trip took longer than planned”.

You do not have other options at this time, but several new rides appear on the website each day, and you have several days to keep looking.

**Trial 2, negative VIB condition**

Please read the following text carefully:

You have to travel long distance next weekend, and the only option to get to your destination is by arranging for a ride with a stranger going the same direction. You create an account on a ride sharing website and find that there is a person going in the desired direction at an acceptable time.

The driver’s name is Ian, he is 23 years old and drives an old sedan. He has mostly positive reviews on his account, for example:

“It was a lot of fun! Ian is a very cool guy, pity the trip didn’t last longer”

But also several negative ones, for instance:

“Ian didn’t drive fast, but he’s very young, and he made a lot of stops and drank energy drinks, he looked really tired. It seemed like he partied before the trip. I was worried he might fall asleep while driving”.

You do not have other options at this time, but several new rides appear on the website each day, and you have several days to keep looking.

**Trial 3, control condition**

Please read the following text carefully:

Yout colleague recommended a professional development course they took, and you are considering enrolling in it.

Judging from the colleague’s description and the online reviews, the course has a lot to do with some of the common tasks that you encounter at work, and the impressions of the course are mostly positive.

**Trial 3, positive VIB condition**

Please read the following text carefully:

Your colleague recommended a professional development course they took, and you are considering enrolling in it.

Judging from the colleague’s description and the online reviews, the instructor of the course requires complete trust in her and her method. She seems to have encyclopedic knowledge of her subject, and she expects students to memorize and understand a lot of material. The course ends with a test in which students are asked to reproduce some of the most important arguments made on the subject.

**Trial 3, negative VIB condition**

Please read the following text carefully:

Your colleague recommended a professional development course they took, and you are considering enrolling in it.

Judging from the colleague’s description and the online reviews, the instructor of the course likes to make students think and asks challenging questions, letting the students figure things out on their own. She doesn’t shy away from controversial topics, and tries to make her class a place for intellectual discussion.
