# Supplementary material for: Social Construction of the Value–Behavior Relation
Source: Front Psychol. 2019 May 1;10:934. doi: 10.3389/fpsyg.2019.00934 (PMC6504687; doi:10.3389/fpsyg.2019.00934)
Supplement: Supplementary file 2 [file Data_Sheet_2.docx]

Appendix B

**Social Construction of the Value-Behavior Relation**

Vladimir Ponizovskiy*, Lusine Grigoryan, Ulrich Kühnen, Klaus Boehnke

*** Correspondence:** Vladimir Ponizovskiy: ponizovskiy@bigsss.uni-bremen.de

Data analysis using raw (uncentered) value scores

Table S1. Simple slope analyses for the effects of value on attitude and behavioral intention in each experimental condition using uncentered value scores

|  | Attitude | | Behavioral Intention | |
| --- | --- | --- | --- | --- |
|  | *B* | SE | *b* | SE |
| Trial 1 |  |  |  |  |
| Positive VIB | .44*** | .05 | .51*** | .06 |
| Negative VIB | -.12* | .06 | -.13^+^ | .08 |
| Control | .31*** | .04 | .36*** | .05 |
| Trial 2 |  |  |  |  |
| Positive VIB | .23*** | .06 | .22** | .07 |
| Negative VIB | -.16** | .06 | -.22** | .08 |
| Control | -.06 | .06 | -.19* | .08 |
| Trial 3 |  |  |  |  |
| Positive VIB | .24*** | .04 | .38*** | .06 |
| Negative VIB | .02 | .04 | .03 | .05 |
| Control | .22*** | .04 | .33*** | .04 |

*Statistical significance levels: ^+^ p < 0 .10, *p < 0.05, **p < 0.01, ***p < 0.001.*

**Data analysis using raw (uncentered) value scores**

Table S2. Summary of analyses of covariance using uncentered value scores

|  | Attitude | | | Behavioral Intention | | |
| --- | --- | --- | --- | --- | --- | --- |
|  | F | *p* | η_p_^2^ | F | *p* | η_p_^2^ |
| Trial 1 |  |  |  |  |  |  |
| Intercept | 314.8 | <.001 | .556 | 355.7 | <.001 | .186 |
| Religiosity | .003 | .955 | <.001 | 4.16 | .042 | .003 |
| Value (UNc) | 49.2 | <.001 | .031 | 52.5 | <.001 | .033 |
| Condition | 4.76 | .009 | .006 | 4.6 | .010 | .006 |
| Value*Condition | 30.1 | <.001 | .037 | 30.5 | <.001 | .038 |
| Trial 2 |  |  |  |  |  |  |
| Intercept | 437.2 | <.001 | .206 | 376.1 | <.001 | .182 |
| Value (SEp) | <.000 | .986 | <.001 | 1.96 | .162 | .001 |
| Condition | 2.05 | .129 | .002 | 1.39 | .249 | .002 |
| Value*Condition | 12.4 | <.001 | .014 | 10.6 | <.001 | .012 |
| Trial 3 |  |  |  |  |  |  |
| Intercept | 1520.7 | <.001 | .470 | 981.9 | <.001 | .364 |
| Value (COr) | 49.2 | <.001 | .028 | 72.5 | <.001 | .041 |
| Condition | 19.6 | <.001 | .022 | 28.5 | <.001 | .032 |
| Value*Condition | 9.73 | <.001 | .011 | 14.5 | <.001 | .017 |
| *UNc (universalism—concern); SEp (security—personal); COr (conformity—rules).* | | | | | | |
